# Book Reviews

**Published:** 1895-08

**Authors:** 


### *BOOK REVIEWS.*

---

IMMUNITY AND PROTECTIVE INOCULATIONS IN INFECTIOUS DISEASES, AND SERUM THERAPY. Geo. M. Sternberg, M.D., LL.D., Surgeon-General U. S. Army, Ex-President American Public Health Association, etc. New York. Wm. Wood & Co. 1895.

In this age of progressiveness and wonderful discoveries in medicine, and especially in biological medicine, important, indeed, is the epitomizing of data thus far procurable. This work gives evidence of the master hand, for within its bindings are the opinions of the best writers, the author's included.

The subject-matter is well chosen, and most succinctly and clearly given. The reader will have, after careful study of this book, learned much that is new and truly scientific. There is nothing in this work to criticise adversely, but much to emphasize as matter of practical value. The chapters on "Natural Immunity," and "Acquired Immunity," "Cholera," "Diphtheria," "Hydrophobia," "Tetanus," "Tuberculosis," and "Typhoid Fever" are most thorough and seldom do you see so much in so limited a space. The chapters on "Anthrax," "Chicken and Hog Cholera," "Foot and Mouth Disease," "Glanders," "Hog Erysipelas," "Influenza," "Pleuro-Pneumonia," "Pneumonia," "Rinder Pest," "Small Pox," "Swine Plague," "Streptococcus Infection," "Black Leg," and "Yellow Fever" are equally interesting and instructive, though possibly of less interest to the general practitioner of medicine.

Continued study in this especial branch of science cannot fail to be productive of even greater good, and I am quite prepared to join with the author in expecting, within a very short time, to see the antitoxins and serums used daily in the practice of immunizing and inoculating against the diseases within treated.

In closing, I take pleasure in recommending this work to all progressive practitioners., The publishers have produced a neat and well wrought volume. H. H.

A MANUAL OF MEDICAL JURISPRUDENCE. By Alfred S. Taylor, M.D., F.R.S. Revised and edited by Thomas Stevenson, M.D., Examiner in Forensic Medicine in the University of London. Edited with citations and additions by Clark Bell, Esq., of the New York bar. Price $4.50. Published by Lea Bros. & Co., 706, 708 Sansom St., Philadephia.

No one knows at what moment he will be called upon to testify in court upon some medico-legal subject, more or less important. Therefore, every well-informed physician should at least be acquainted with the general principles of medical jurisprudence; and his library should by all means contain some standard work on legal medicine.

Taylor's Medical Jurisprudence long ago earned its right to be called a standard work; it is so recognized by both doctors and lawyers. The several revisions and editions through which it has passed have only added to its original value. This edition discusses in a masterly manner the usual subjects embraced in the name medical jurisprudence, and that the work is brought well down to date is seen in the chapters relating to electrocution, the detection of blood stains, ptomaine poisoning, etc. The fine hand of Mr. Bell is seen throughout the book wherever the text can be rendered more useful for the American reader, and his numerous citations of illustrative American cases greatly increase its value as a reference book for both professions.

There are other and more exhaustive works on legal medicine, but for a safe, practical, and thoroughly reliable manual on this subject, the present work is everything that could be desired, and we cheerfully recommend it.

---

A TREATISE ON THE NERVOUS DISEASES OF CHILDREN. For Physicians and Students. By B. Sachs, M.D., Professor of Nervous and Mental Diseases in the New York Polyclinic; Consulting Neurologist to the Mt. Sinai Hospital, etc., etc. New York. Wm. Wood & Co. 1895.

In reviewing a work, it is not always an easy task to express one's opinion, especially when one is favorably impressed, for, in all probability, our praise will be marked by fulsomeness. Dr.

Sachs has given to the profession a much needed treatise. A gap has been bridged which will enable many practitioners to intelligently diagnose and treat cases heretofore beyond their ken.

The author has evidently given his subject-matter great study, and has succeeded in winnowing from the mass of litueratnre on these subjects all that is good, and just up to date. The study of this volume is a great pleasure, for the reader is not depressed by reiteration and offended by the ordinary compilation.

The cuts, diagrams, and plates are good. The subjects are well arranged and classified. It would take much space to criticise especial chapters and subjects, and then we could not say more than that the book is in every respect admirable, as far as neurological knowledge extends, and fully deserving the study of any man.

Messrs. Wm. Wood & Co. have displayed their usual artistic and exquisite workmanship in its printing and binding. H. H.

---

THE CARE OF THE BABY. A Manual for Mothers and Nurses. By J. P. Crozer Griffith, M.D., Clinical Professor of Diseases of Children in the Hospital of the University of Pennsylvania, Physician to the Children's Hospital, etc. Illustrated. W. B. Saunders, 925 Walnut Street, Philadelphia.

This book fully sustains its title in being a simple, clear, and practical manual for mothers and nurses. The young woman in her first pregnancy will find this little volume to abound in the most valuable information and advice on an important subject too often neglected by mothers. The several chapters relate to the hygiene of pregnancy, the characteristics of a healthy baby, suggestions as to the baby's clothing, bathing, feeding, physical exercise, and training, the hours for nursing and the hours for sleeping, etc. The indications and symptoms of the more common diseases of infancy are touched upon briefly, with some suggestions as to the management of the most simple troubles, etc. Some useful formulæ and directions for making and using poultices, hot and cold applications, nutrient broths, sterilized foods, etc., close the volume. The book is about the best of its kind we have seen. It is not so simple, either, but the practitioner of medicine, young or old, will find it useful and instructive.

